# Diagnostic Application of IS*900* PCR Using Blood as a Source Sample for the Detection of *Mycobacterium avium* Subspecies *Paratuberculosis* in Early and Subclinical Cases of Caprine Paratuberculosis

**DOI:** 10.4061/2010/748621

**Published:** 2009-11-16

**Authors:** P. K. Singh, S. V. Singh, H. Kumar, J. S. Sohal, A. V. Singh

**Affiliations:** Microbiology Laboratory, Animal Health Division, Central Institute for Research on Goats, Makhdoom, PO - Farah, Mathura (UP), Uttar Pradesh 281 122, India

## Abstract

Efficacy of IS*900* blood PCR was evaluated for the presence of MAP infection. Serum, fecal, and blood samples of kids, young, and adult goats from farm and farmer's herds in Mathura district were also screened by ELISA, microscopy and culture. Of 111 goats (kids: 40, young: 14, adults: 57) screened, 77.5% were positive by blood PCR. Of 76 goats, 90.8% (kids: 87.5% and adults: 94.4%) were positive by PCR. From 21 kids and 14 young goats, 42.8 and 57.1% were positive. gDNA from goats was genotyped as MAP “Indian Bison type”. Of 21 fecal samples of kids examined by microscopy, 66.7% were positive. In ELISA, 9.5 and 57.1% kids were positives as “type I” and “type II” reactors, respectively. Screening 14 young goats by culture of blood clots, 28.6% were positive. Agreement was substantial between PCR and microscopy. It was fair and moderate when PCR and microscopy were compared with type I and type II reactors, respectively. Presence of MAP in non-clinical kids and young goats indicate early or subclinical infection. Blood PCR was rapid, sensitive, and specific assay for detection of MAP in any stage (early, subclinical, and clinical) and age (kids, young, and adult) of goats.

## 1. Introduction

Johne's disease (JD) caused by *Mycobacterium avium* subsp. *paratuberculosis* (MAP) is responsible for huge losses in production [1]. MAP has impact on food safety and also associated with Crohn's disease [2] in humans. Incubation is long and variable before manifestation of clinical signs [3]. JD was endemic in farms and farmer's herds located in Mathura district [2, 4, 5]. However, information in young kids is limited [6]. Kids get infected via milk and in utero [7, 8]. Following oral infection, MAP invade intestinal macrophages [9] and clinical disease has been reported in young kids [7, 10]. Subclinically infected kids (carriers) continue to shed MAP before converting to a clinical case in adulthood. Therefore, diagnosis of MAP infection in kids and young goats is crucial for the control of disease.

Fecal culture is widely accepted as the most reliable in the clinical stage [11] but is time consuming [12]. Sensitivity of culture in subclinical stage is low and depends heavily on shedding of MAP in feces. Isolation of MAP from sites distant from intestines such as udder, fetus, kidney, liver, and male reproductive tract [7, 13] suggests active dissemination of MAP in milk, semen, and transplacental infection of fetuses, establishing continuous movement of MAP in the blood stream.

ELISA, though a popular screening test, suffers from low sensitivity in early and subclinical phase specially in young kids [2, 14]. However, utility of serology is compromised by antibodies rarely produced at detectable levels in early stages of infection. In whole-herd testing sensitivity of serologic assays is less than 50% as compared to fecal culture. Detection of MAP by IS*900* PCR in fecal samples though rapid but is low throughput in kids as MAP shedding at early to subclinical stage is rare or intermittent and also due to the presence of PCR inhibitors [14]. After infection, MAP (within monocyte) circulate via blood stream to various organs, therefore, detection of MAP by IS*900 *PCR in blood samples may help in diagnosis of JD in young animals and chances of detection of false positive (due to passive infection) will be zero or low. Recently, IS*900* PCR-based detection of MAP from white blood cells (WBCs) has been described [15, 16], but use in animals is still limited [17]. IS*900* elements have also been reported from mycobacteria other than MAP [18]. PCR assays using primers specific for *F57*, IS*Mav2*, IS*MAP02*, and IS*MAP04 *elements have been used for specific detection of MAP DNA [19–22]. However, the higher number of copies of IS*900* element in comparison to other IS elements makes IS*900-*based detection very sensitive.

The present study aimed to determine efficacy of IS*900 *using blood as source samples (blood PCR) for the diagnosis of JD in early and subclinical stages in kids, young, and adult goats. Blood PCR was also evaluated with ELISA, microscopic examination, and blood culture for the detection of MAP in goats from different age groups, stage of disease, and farm and farmer's herds endemic for JD.

## 2. Materials and Methods

### 2.1. Animals and Samples

Goats (kids and adults) of two important Indian breeds Jamunapri (60) and Barbari (51) belonging to two sources were screened for MAP infection. Goats from the government farm (Central Institute for Research on Goats (CIRG), Mathura district, Uttar Pradesh) and farmer's herds (Mathura district, Uttar Pradesh) were named “source A” and “source B,” respectively, where JD was endemic [2]. Blood, serum, and feces of 21 Barbari kids (3-4 months) from “source B” were screened using blood PCR, ELISA, and microscopic examination, respectively. Serum was harvested from 21 kids by clotting part of blood samples and clots were processed for culture. Blood and serum of 14 male Barbari goats (10–12 months) of “source A” were collected before being sacrificed as part of another experiment. The 76 goats (40 kids and 36 adult) of Jamunapari (60) and Barbari (16) breed from “source A” were screened by Blood PCR, where JD was endemic since the establishment of these farms [23, 24]. Blood samples (500 *μ*L) were collected in Eppendorfs containing 50 *μ*l of 2.7% EDTA from each of 111 goats.

### 2.2. Detection of MAP

#### 2.2.1. Isolation of DNA and Blood PCR

One milliliter of erythrocyte lysis buffer (320 mM Dextrose, 5 mM MgCl_2_, 1% triton X-100, Tris HCl 10 mM; pH-7.5) was added to 500 *μ*l blood samples. Tubes were vortexed vigorously and spun at 15000 g for 2 minuntes. Pellet containing WBCs was again treated with erythrocyte lysis buffer until the pellet became white. The 400 *μ*l of nucleic lysis buffer (60 mM NH_4_Cl; 24 mM Na_2 _EDTA; 1 mg/mL Proteinase K; pH-8) and 100 *μ*l of 1% SDS were mixed and used to suspend the WBC pellet and incubated at 55°C in a water bath for 30 minutes. After digestion samples were cooled at room temperature and centrifuged at 15000 g for 10 minutes. Supernatant was collected and 100 *μ*l of ammonium acetate (3 M) was added and again centrifuged at 15000 rpm for 10 minutes. Supernatant containing genomic DNA of goats and MAP DNA (if present) was transferred to fresh eppendorf. A double volume of absolute ethanol was added and the tubes were gently inverted several times until DNA threads were precipitated. Tubes were centrifuged at 15000 g for 10 minutes. The DNA pellet was washed with 1 mL of 70% ethanol, air dried, resuspended in 30 *μ*l of TE buffer (pH 8), and kept at −20°C for further use.

MAP specific primers unique to MAP (IS*900* P 90/91) as per Miller et al. [25] were procured. Primers sequences used were

- forward primer- P90B 5′-GAA GGG TGT TCG GGG CCG TCG CTT AGG -3′
- reverse primer- P91B 5′-GGC GTT GAG GTC GAT CGC CCA CGT GAC -3′

Red dye master mix kit (Bangalore Genei, Bangalore) containing all components of reaction mixture (dNTPs, *Taq *polymerase, Assay buffer and MgCl_2_, loading dye) was used. The reaction volume was 50 *μ*l containing 5 *μ*l (100–200 ng) of test DNA sample, 1 *μ*l of each primer (10 pico-mole). Reaction mixtures containing positive (DNA from native “Bison type” S 5 strain of MAP) and negative (sterilized liquipure water) controls were also thermocycled. Briefly the reaction conditions were 94°C, 3 minutes (initial denaturation) for one cycle, 94°C, 30 seconds (denaturation), 63°C, 15 seconds (annealing), 72°C, 1 minutes (extension) for 30 cycles and a final extension of 72°C, 10 minutes for 1 cycle and stored at 4°C. The PCR product was analyzed on a 1% agarose gel in 1XTBE buffer containing 0.5 *μ*g/mL of ethidium bromide at 80 V for 1 hour. Known positive amplified product and gene ruler DNA ladder plus 100 bp (MBI, Fermentas) were also run. Gels were visualized using the Gel document system, Alpha Innotech.

#### 2.2.2. Culture (Blood Clots)

Blood clots were cultured as per Singh et al. [4] with few modifications. MAP isolates from Mathura were “Bison type” [26] and Herrold's Egg Yolk (HEY) medium without sodium pyruvate was used. Clots were crushed in 3-4 mL sterilized NSS/PBS and transferred to a fresh tube for overnight sedimentation. Five mL of supernatant was decontaminated in 0.9% Hexadecyl pyridinium chloride (HPC), for 18–24 hours at room temperature. About 0.2 mL of sediment was inoculated on HEYM slants, incubated at 37°C for 18 weeks, and observed weekly. MAP colonies were identified on the basis of appearance time (slow growing), colony morphology, acid fastness, cellular morphology, and mycobactin J dependency.

### 2.3. Genotyping of MAP Infection by IS1311 PCR-REA

IS*1311* PCR was carried out using M56 and M119 primers as per Sevilla et al. [26]. Briefly, each PCR was set up in a 25 *μ*L volume, using 0.5–1.0 ng template DNA, 2.5 *μ*l of 10X PCR buffer (Promega), 1.5 mM MgCl_2 _(Promega), 0.2 mM dNTPs, and 1 unit *Taq* (Promega). Cycling conditions were an initial denaturation at 94°C for 3 minutes followed by 37 cycles of denaturation at 94°C for 30 seconds, annealing 62°C for 30 seconds and an extension at 72°C for 1 minute followed by a final extension at 72°C for 10 minutes. An amplicon size of 608 bp was interpreted as positive for IS*1311* PCR, after separation on 2% agarose gel stained with ethidium bromide.

IS*1311* PCR-REA was also carried out as per Sevilla et al. [26]. Briefly, the reaction was carried out in a 30 *μ*l volume, containing 20 *μ*l positive IS*1311* PCR product, 3 *μ*l 10X buffer (Fermentas), and 2 units of each endonuclease *Hinf* I and *Mse* I (Fermentas). Reaction mixture was incubated at 37°C for 1.5 hours, and patterns were visualized and compared with the pattern of “Cattle type”, “Sheep type”, “Bison type”, and *M. avium* after electrophoresis on 4% agarose gel stained with ethidium bromide.

### 2.4. Microscopic Examination of Ziehl Neelsen Staing Fecal Smear

About 2 gm of fecal sample was homogenized in 3-4 mL of sterilized normal saline solution (NSS) in pestle mortar and made into a fine paste. This paste was transferred to 15 mL centrifuge tubes after diluting with 7-8 mL of sterilized NSS. The solution was centrifuged at 4000 rpm for 45 minutes to concentrate bacilli. Following centrifugation, the top layer was decanted, the semisolid middle layer was collected by loop, and a thin layer smear was made over the glass slide. Smear was heat fixed and stained with Ziehl Neelsen's stain and visualized under the microscope for pink colored small rods.

### 2.5. ELISA Test

Goats were screened by “indigenous ELISA kit” [2]. Semipurified protoplasmic antigen (PA) was prepared from MAP S 5 (“Indian Bison type” MAP) of goat origin [26, 27] obtained from the Microbiology Laboratory of CIRG, Mathura. Culture was inactivated at 72°C for 2 hours, pelleted at 10000 g for 20 minutes at 4°C, suspended in 0.01 M PBS (pH 7.2), and washed three times. The pellet was finally suspended in NSS at a ratio of 200 mg wet cell/mL and was exposed to ultrasonic disruption (100 watts/15 Hz for 20 minutes). The sonicate was centrifuged at 10000 rpm for 30 minutes at 4°C, and the supernatant was dispensed in 0.5–1 mL aliquots and stored at −20°C. Protein was measured by Lowry et al. [28] method. Antigen, rabbit antigoat horseradish peroxidase conjugate (Banglore Genei, Bangalore), and OPD substrate were used at 0.1 *μ*g/well, 1 : 8000 dilution, and 5 mg/plate, respectively. Sample-to-positive (S/P) ratios (*Negative* 0.00–0.09, *Suspected* or *Borderline* 0.10–0.24, *Low positive* 0.25–0.39, *Positive* 0.40–0.99, *Strong positive* 1.00–10.0) were calculated as per Collins [29]. Serum from a culture positive goat with clinical JD was the positive control, and a culture negative goat was used as the negative control.

ELISA results categorized as *strong positive* were identified as “type I” reactors while those categorized as *strong positives and positives* were identified as “type II” reactors. Sensitivity and specificity of ELISA kits were calculated with respect to blood PCR using the method of Arizmendi and Grimes [30]. Performance of “blood PCR” was compared with indigenous ELISA, microscopic examination, and blood culture by calculating “Kappa Scores” (Proportional Agreement) as per method of Landis and Koch [31] (0<, *poor*; 0.0–0.20, *slight*; 0.21–0.40, *fair*; 0.41–0.60, *moderate*; 0.61–0.80, *substantial* and 0.81–100, *almost perfect*). Performance of indigenous ELISA was compared in our earlier study [32] with commercial kit and was superior.

## 3. Results

### 3.1. Detection of MAP by IS900 PCR

**Figure 1:**
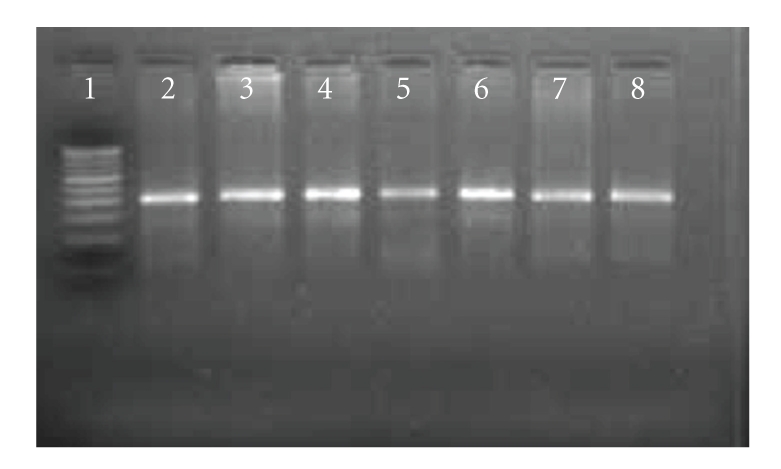
*Mycobacterium avium* subsp. *paratuberculosis* specific amplicons (413 bp) by PCR using IS*900* specific primers. Lane 1: 100 bp DNA ladder, Lane 2: Positive control; Lane 3–8: tested DNA samples.

Positive PCR products using specific IS*900 *primers were detected as a 413 bp product (Figure 1). Of the total 111 goats (Jamunapari and Barbari breed) screened, 77.5% were positive in “blood PCR.” Of the 21 kids from “source B”, 9 (42.8%) were positive for the presence of MAP DNA in the blood samples (Table 1). From the 14 young goats (source A) sacrificed, 8 (57.8%) were positives. Whereas, of 76 farm goats 69 (90.8%) were positives (87.5% in kids and 94.4% in adult goats) by “blood PCR.”

### 3.2. Genotyping of MAP

**Figure 2:**
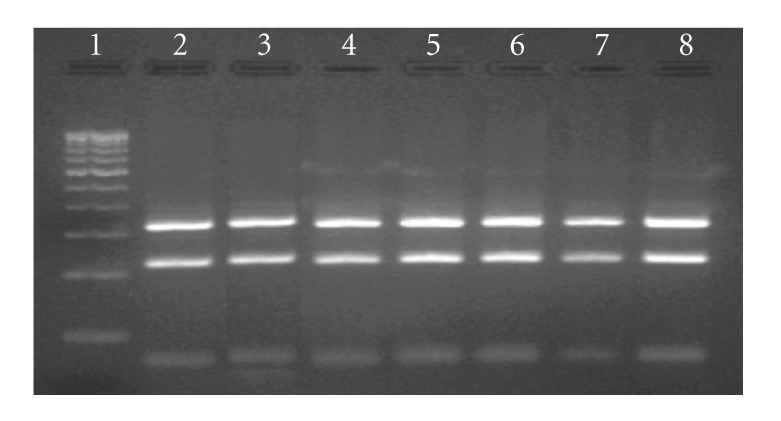
IS*1311* PCR-REA profile (Bison type) of tested samples. Lane 1: 100 bp DNA ladder, Lane 2: control Bison type (S-5 strain) MAP, Lane 3–8: tested samples of different goats (all were “Bison type” of MAP genotype).

DNA of 42.8% positive kids (“blood PCR”) from “source B” was genotyped by IS*1311* PCR-REA. Positive PCR products using specific IS*1311* primers were detected as a 608 bp product. PCR products were intact bands without primer diamer and non-specific amplicons and were suitable for direct restriction digestion without purifying the PCR products. IS*1311* PCR-REA fingerprints developed by digesting the PCR DNA with *Hinf* I and *Mse* I revealed an “Indian Bison type” pattern in all samples similar to the positive control (MAP S-5 strain of the “Indian Bison type” genotype) (Figure 2).

### 3.3. Detection of MAP by Microscopic Examination

Screening of 21 fecal samples of Barbari kids (source B) by microscopic examination, revealed 66.7% positive for MAP infection (Table 1).

### 3.4. Detection of Anti-MAP Antibodies by ELISA

Of the 21 Barbari kids (source B) screened, 9.5, 47.6, 28.6, 0, and 14.3% were in strong positive, positive, low positive, suspected and negative categories of S/P ratios, respectively (Table 2). Only 9.5% of the kids were positive as “type I” reactors (Table 1), however, 57.1% (12/21) kids were positives as “type II” reactors (Table 1).

### 3.5. Detection of MAP by Blood Culture

**Figure 3:**
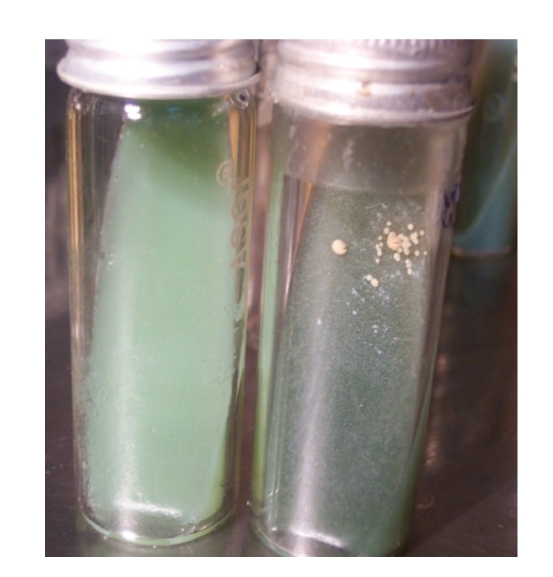
Characteristic colonies of *Mycobacterium avium* subsp. *paratuberculosis *on HEY medium. Colonies appeared only on HEY slants supplemented with mycobactin J (Tube at right side) while absent in duplicate HEY slant (without Mycobactin J—Tube at left side).

Of the 14 blood clots from young male Barbari goats “source A” on screening by culture, 28.6% were positive (Figure 3).

Sensitivity and specificity of indigenous ELISA kits with respect to “blood PCR” was 6.2 and 80.0% and 56.2 and 40.0% as “type I” and “type II” reactors, respectively.

### 3.6. Comparison of Tests

Proportional agreement (PA value) between “blood PCR” and microscopic examination was substantial (71.0%). When “blood-PCR” and microscopic examination were compared with “type I” reactors, the PA values were 23.0 and 33.3% (fair), respectively. Whereas in “type II” reactors, PA value with respect to “blood PCR” and microscopic examination were 52.0% (moderate) each.

## 4. Discussion

Early diagnosis of Johne's disease (JD) is crucial for the control of disease in herds. Widely reported studies on clinical JD with respect to bacteriology, immunology, histology and their relationships [33–35] did not provide information on septicemia and time by which MAP is disseminated to blood stream. JD challenge models for various species has been proposed but time of onset of infection to appearance of MAP in blood has also not been predicted. MAP being intracellular is likely to be disseminated by blood phagocytes [36]. It is assumed that MAP septicemia occurs in subclinical and mainly in the clinical stage [37, 38]. Current diagnostic tests lack 100% sensitivity and specificity and ability to detect infection at early stages or in young animals [39]. Considering PCR as rapid and powerful tool to specifically probe and amplify DNA of MAP, a significant proportion of sheep with advanced clinical JD were detected by using PCR in blood samples [40]. PCR using blood as the source sample reduced the possibilities of detecting passive infection. The test raised hopes for detecting subclinical MAP infection. In the present study, goats were screened using IS*900* PCR on DNA (extracted from blood) to obtain the frequency of distribution of MAP in young kids and adult goats of “source A and B”. PCR was also compared with ELISA, microscopy examination of fecal samples and blood culture on a small number of kids and young goats. Though, Englund et al. [18] reported IS*900* like elements in other mycobacteria, in the present study IS*900* PCR was used due to higher sensitivity and presence of a greater number of IS*900* copies than other MAP specific IS elements. Moreover, in the present study MAP specific IS*1311* PCR-REA has been carried out as confirmatory test for MAP and positive goats of “source B” were genotyped as “Indian Bison type”.

In this study, 111 kids and adult goats from endemic herds were screened for MAP septicemia by blood PCR and a very high (77.5%) MAP septicemia was reported in this study. MAP infection was moderate (42.8%) in “source B” as compared to “source A” where it was high (87.5%). In the “source A” herds, infection was moderate (57.8%) in young goats sacrificed after feedlot studies as compared to adult goats (94.4%), since MAP infection was endemic in the farm herds [4, 5, 8] screened.

JD is a chronic disease and clinical symptoms generally appear after long (2-3 years) subclinical phase. Since positive kids were young and did not show clinical symptoms, it may be assumed that kids were in early subclinical stage of infection. Therefore, the present study challenged the general concept that MAP septicemia occurs in subclinical to clinical stage of disease, though infection rate/septicemia was highest in clinically infected adult goats (94.4%). To conclusively prove infection, blood clots of 14 young male Barbari goats (source A) were simultaneous cultured and viable MAP were recovered from blood samples of 28.6% goats by culture whereas, 57.8% were detected by “blood PCR”. Whipple et al. [41] also reported PCR to be more sensitive than culture. Characteristic MAP colonies obtained in culture confirmed septicemia of MAP.

Though conventionally infection occurs through intestinal route, recently tonsils have been reported as an alternative port of entry for MAP when dose of infection is high [6]. It is also believed that infection through the tonsil port may be the shortest route to enter in to the blood stream. JD was endemic in Mathura region and the load of MAP in the environment is very high and a high dose of MAP daily may allow the pathogen to follow the tonsilar route of infection and may be an important reason for the high presence of MAP in the blood of kids and young goats in this region.

It is reported that the chances of transplacental infection increases up to 12% in subclinically infected animals [42] and higher (20 to 40%), in clinically infected animals [43] and making control of the disease difficult at herd level. High presence of MAP in young goats in endemic regions like Mathura also reflected the possibilities of trans-placental transmission of MAP. Of the 36 adults goats 94.4% were positive by “blood PCR”. Few positive adults (6) exhibited clinical symptoms of JD whereas others were apparently normal but not healthy (low growth rate and low feed conversion efficiency).

High rate of MAP infection in these goats may also be attributed due to higher susceptibility of Barbari and Jamunapari breed of goats to MAP infection [23, 24]. Genotyping of MAP DNA revealed that all were “Indian Bison type”, a highly pathogenic [10, 44] and most prevalent genotype in Northern India [5, 26]. Interaction between susceptible breeds (Barbari and Jamunapari) with highly pathogenic MAP genotype (“Indian Bison type”) in an endemic environment led to high recovery of MAP from blood samples.

Gwozdz et al. [37] contrarily showed poor performance of “blood PCR” to detect subclinically infected sheep. Of 117 samples of blood sequentially collected over 53 weeks from 14 experimentally challenged sheep, only two samples were positive. Poor detection may be due to less severe extra intestinal infection in challenged sheep or improved optimization of “blood-PCR” in naturally infected goats in the present study or higher levels of infections. Barrington et al. [38] had also recorded lower sensitivity of “blood-PCR” in comparison to PCR applied on milk, liver and fecal samples of advanced subclinically infected cows. Isolation of MAP from extra intestinal locations indicate sporadic bacteraemia resulting from either direct invasion of blood vessels by the bacilli or access to circulation through draining lymphatics, lymph nodes and thoracic ducts [45].

“Blood-PCR” was used to detect MAP due to difficulties encountered in growing MAP isolates in-vitro (by culture). In many studies, a PCR assay was applied on DNA extracted from peripheral blood mononuclear cells (PBMCs) isolated from 5–10 mL of blood. Isolation of PBMCs from whole blood is costly and required a greater amount of blood and attention. However, in the present study a simple method of DNA isolation was standardized which required only 500 *μ*l of blood and was cost effective and user friendly and may be adopted for human samples a well. Along with “blood-PCR”, ELISA and microscopy were used on 21 male kids of Barbari breed. Of these, “blood PCR” was most sensitive followed by direct microscopy and ELISA (type II reactors) to detect MAP in young goats. In ELISA, 9.5 and 57.1% kids were positive in “type I” and “type II” reactors, respectively. Animals in the early stages of infection often do not elicit detectable immune responses by currently available tests [34, 46]. This may be attributed to low sensitivity of ELISA. With respect to PCR, sensitivity and specificity of ELISA was 6.2 and 80.0% and 56.2 and 40.0% in “type I” and “type II” reactors, respectively.

In the present study the different test were compared using kappa statistics. Though kappa statistics is popular in comparing the efficiency of different tests, Kappa score calculations and their resulting interpretation for agreement between tests is not universally accepted. Agreement implies only that the two tests are measuring the same or closely correlated factors. Therefore, good agreement does not necessarily imply correctness of test results relative to infection. As a caution, MacLure and Willett [47] noted that the kappa statistic was originally proposed as a measure of reproducibility, and that sensitivity and specificity represent better measures of test validity than does kappa. Also, MacLure and Willett [47] challenged the use of significance testing of kappas to assess the degree of agreement. The sensitivity and specificity of different diagnostics (used in diagnosis of JD) depend on the stage/level of infection; therefore there may be chance to misinterpretation of the agreement between different tests. Despite of the limitations of kappa statistics has been used in many earlier studies [48–50] and also in the present study as supportive information regarding agreement between tests. In “type I” reactors, ELISA had *fair* proportional agreement (23% and 33.3%) both with “blood PCR” and microscopy. Whereas, “type II” reactors had *moderate* proportional agreement (52%) both with “blood PCR” and direct microscopy. However, “blood PCR” and direct microscopy had substantial correlation between the two. Comparison of 3 tests revealed (Tables 1 and 2), that only 2 animals from 21 screened were true negatives. The remaining 19 were positive in 3, 2, and/or single test combinations.

Efficacy of a diagnostic test for MAP infected herds depends on the frequency of testing the individual animals at each stage of the disease [51]. Subclinically infected animals represent a reservoir for MAP in a herd. In order to validate the accuracy of PCR detection of early and subclinical goats, more goats would be needed for the screening of hematogenous spread of MAP and also a longitudinal study followed by necropsy. These tests should also be compared with fecal culture, ELISA and microscopic examination of the same samples. Stage of JD greatly influences the sensitivity of test. The present study indicated that detection of MAP DNA as a measure of infection is possible before the animals develop a positive sero-status in kids. Goats identified by PCR may be in an early to subclinical phase of infection. In kids, absence of JD symptoms (except in 2 goats) also supports that infection was of an early subclinical type. PCR on blood samples seemed to be a potential diagnostic tool which may be used to screen young kids as well as other animals in early to subclinical stages of infection. PCR had a higher degree of predictability for the detection of MAP when compared with ELISA and microscopic examination of fecal smears in young goats. Increased sensitivity of PCR using blood samples may be also due to detection of both viable and nonviable bacteria. High presence of MAP infection in young kids correlated well with the endemicity of the MAP infection in the herds under study [5, 8].

## 5. Conclusions

“Blood PCR” was rapid, highly sensitive, and specific for detecting MAP infection in kids, young, and adult goats. Prevalence of MAP in farm (source A) and farmer's (source B) herds was high.

## Figures and Tables

**Table 1:** Evaluation of ELISA with blood-PCR and microscopic examination.

S/P ratios	Johne's disease status	Number (%)	Positives	
			Blood PCR	ME*
00.0–0.9	Negative	03 (14.3)	1	1
0.1–0.24	Suspected	00 (00.0)	—	—
0.25–0.39	Low Positive	06 (28.6)	2	5
0.4–0.9	Positive	10 (47.6)	5	7
1.0–10.0	Strong Positive	02 (09.5)	1	1
Total			9 (42.8)	14 (66.7)

*ME: microscopic examination.

**Table 2:** Comparison of blood-PCR with ELISA (type I and type II reactors) and microscopic examination for the detection of *Mycobacterium avium* subsp. *Paratuberculosis* infection in kids.

Tests	Combinations							
	1	2	3	4	5	6	7	8
PCR	+	−	+	−	−	+	−	+
ME	+	−	−	+	−	+	+	−
ELISA	+	−	−	−	+	−	+	+
Total (A)	1	2	4	2	1	11	0	0
Total (B)	6	2	1	0	1	6	2	3

(A) represent the total number of positive animals in different diagnostic combinations and type I reactor was considered as positive in ELISA.

(B) represent the total number of positive animals in different diagnostic combinations and type II reactor was considered as positive in ELISA.
